# Towards a More Comprehensive Picture of the MicroRNA-23a/b-3p Impact on Impaired Male Fertility

**DOI:** 10.3390/biology12060800

**Published:** 2023-05-31

**Authors:** Lea Simone Becker, Mohammad A. Al Smadi, Hanna Koch, Hashim Abdul-Khaliq, Eckart Meese, Masood Abu-Halima

**Affiliations:** 1 Institute of Human Genetics, Saarland University, 66421 Homburg, Germany; 2 Reproductive Endocrinology and IVF Unit, King Hussein Medical Centre, Amman 11855, Jordan; 3 Department of Pediatric Cardiology, Saarland University Medical Center, 66421 Homburg, Germany

**Keywords:** male subfertility, oligoasthenozoospermia, miRNA-target-interaction, expression level, microRNA-23a/b-3p

## Abstract

**Simple Summary:**

This study focused on the relationship between microRNA-23a/b-3p and genes involved in human spermatogenesis, with the aim of understanding how this microRNA targets these genes and how it affects male fertility. Through computational prediction and experimental assays, we identified specific genes that are targeted by microRNA-23a/b-3p. By intentionally modifying the binding sites of microRNA-23a/b-3p in these genes, we confirmed the direct targeting. To validate their findings, we analyzed sperm samples from men with low sperm count and motility compared to a control group. The results revealed that the men with fertility issues had lower expression levels of the target genes. Furthermore, we found a positive correlation between basic semen parameters and the reduced expression levels of the target genes. This indicates that microRNA-23a/b-3p plays a significant role in spermatogenesis by regulating the expression of target genes associated with male fertility problems, and it also has an impact on basic semen parameters. Overall, this study provides important insights into the involvement of microRNA-23a/b-3p in spermatogenesis and its potential influence on male fertility.

**Abstract:**

The expression levels of various genes involved in human spermatogenesis are influenced by microRNAs (miRNAs), specifically microRNA-23a/b-3p. While certain genes are essential for spermatogenesis and male germ cell function, the regulation of their expression remains unclear. This study aimed to investigate whether microRNA-23a/b-3p targets genes involved in spermatogenesis and the impact of this targeting on the expression levels of these genes in males with impaired fertility. In-silico prediction and dual-luciferase assays were used to determine the potential connections between microRNA-23a/b-3p overexpression and reduced expression levels of 16 target genes. Reverse transcription-quantitative PCR (RT-qPCR) was conducted on 41 oligoasthenozoospermic men receiving infertility treatment and 41 age-matched normozoospermic individuals to verify the lower expression level of target genes. By employing dual-luciferase assays, microRNA-23a-3p was found to directly target eight genes, namely *NOL4, SOX6, GOLGA6C, PCDHA9, G2E3, ZNF695, CEP41*, and *RGPD1*, while microRNA-23b-3p directly targeted three genes, namely SOX6, *GOLGA6C*, and *ZNF695*. The intentional alteration of the microRNA-23a/b binding site within the 3′ untranslated regions (3′UTRs) of the eight genes resulted in the loss of responsiveness to microRNA-23a/b-3p. This confirmed that *NOL4, SOX6, GOLGA6C, PCDHA9*, and *CEP41* are direct targets for microRNA-23a-3p, while *NOL4, SOX6*, and *PCDHA9* are direct targets for microRNA-23b-3p. The sperm samples of oligoasthenozoospermic men had lower expression levels of target genes than age-matched normozoospermic men. Correlation analysis indicated a positive correlation between basic semen parameters and lower expression levels of target genes. The study suggests that microRNA-23a/b-3p plays a significant role in spermatogenesis by controlling the expression of target genes linked to males with impaired fertility and has an impact on basic semen parameters.

## 1. Introduction

MicroRNAs (miRNAs) have become a prominent field of genetic research since their discovery, owing to their significant role in regulating gene expression post-transcriptionally. These small, non-coding RNAs, which are approximately 22 nucleotides in length, primarily function through complementarity with the 3′ untranslated region (3’UTR) of the target messenger RNA (mRNA) [1]. MiRNAs play a vital role in numerous cellular and molecular processes, including development, cell differentiation, and physiological homeostasis [2]. It is, therefore, not unexpected that the aberrant expression of certain miRNAs can disrupt critical processes and lead to various pathologies [3]. Moreover, miRNAs’ disease-specific expression patterns make them suitable prognostic biomarkers [4]. As of today, 2300 mature human miRNAs have been validated [5], and more than 60% of protein-coding genes are predicted to contain target sites for miRNA binding [6]. In the case of humans, miRTarBase, a database for experimentally validated microRNA–target interactions, has documented over 17,000 target genes and more than 4 million miRNA–target interactions (MTIs) [7]. However, the majority of predicted MTIs have yet to be studied experimentally, despite the high number of confirmed target genes. MiRNA has been identified as a crucial regulator during different stages of spermatogenesis and early embryonic development in male human reproduction, as reviewed by Salas-Huetos et al. [8]. Additionally, the involvement of miRNAs in male infertility development is widely accepted, with observed alterations in miRNA expression levels in human sperm, seminal plasma, and testicular tissue of men with various patterns of non-obstructive azoospermia [8]. In recent studies, a correlation has been observed between the differential expression of miRNAs and/or their target mRNAs levels, and basic semen parameters, notably sperm motility [9,10]. This correlation suggests that miRNAs and/or their target mRNAs can potentially serve as diagnostic, prognostic, and even therapeutic biomarkers for male infertility. Specifically, higher expression levels of microRNA-23a/b-3p have been linked to lower expression of a group of testis-specific target genes in male patients with subfertility [11,12], which helps in comprehending the complexity of miRNA regulation during spermatogenesis and/or sperm function. However, although prediction tools for MTIs are continually improving, they still lack the ability to confidently validate miRNA target genes. Therefore, the development and improvement of algorithms rely on experimental validation and expanding the verified target genes is of utmost importance. This study aims to experimentally validate a possible connection between the level of microRNA-23a/b-3p and another set of 16 testis-specific targets using dual-luciferase assays and RT-qPCR validation in human spermatozoa from healthy and oligoasthenozoospermic individuals.

## 2. Materials and Methods

### 2.1. Ethics Approval

The study adhered to the principles of the Declaration of Helsinki and received approval from the Saarland Medical Association Ethical Board (Ha 195/11/updated June 2021). All participants, including both patients and healthy donors, provided written and informed consent before participating in the study.

### 2.2. In-Silico Prediction of MicroRNA-23a/b-3p Target Genes 

The miRWalk algorithm 2.0 was utilized for an in silico analysis to identify potential target genes of microRNA-23a/b-3p [13]. To narrow down the selection, only those target genes predicted in at least 4 out of 12 prediction algorithms incorporated into the miRWalk algorithm were considered, which yielded around 13,000 potential target genes. Further refinement was done by cross-matching these predicted target genes with genes specifically expressed in the testes, based on the Human Protein Atlas (proteinatlas.org, 2237 genes), resulting in 1097 potential target genes [14]. These target genes, which were identified based on the miRWalk algorithm and the Human Protein Atlas, respectively, were found to bear a microRNA-23a/b-3p and play a role in spermatogenesis. Among the 1097 potential target genes, 16 target genes namely *NOL4, SOX6, GOLGA6C, PDCHA9, G2E3, ZNF695, CEP41, RGPD1, GOLGA6B, LMLN, ZNF492, GNAT1, REEP1, CSNK1G1, FAM169A,* and *TMEM215* were selected for further investigation.

### 2.3. Cloning of MiRNA Expression and 3’UTR Reporter Vector Constructs

In order to overexpress microRNA-23a/b-3p, expression vectors pSG5-miR-23a-3p and pSG5-miR-23b-3p were employed. The overexpression of microRNA-23a/b-3p using the pSG5 expression vector was confirmed by Northern blotting [11]. To generate pMIR-RNL-TK reporter vectors containing the 3’UTR of the chosen 16 target genes, a cloning procedure was performed. This involved amplifying and inserting 3’UTR fragments from the genomic DNA template into the multiple cloning site (MCS) downstream of the luciferase gene. This was achieved using sequence-specific forward and reverse primers, which had an additional 5′ SpeI (5′-ACTAGT-3′) and SacI (5′-GAGCTC-3′) restriction enzyme cleavage site, as outlined in Table S1. Mutagenesis was carried out to modify the binding sites within the 3’UTR of vector constructs by using overlap extension PCR and specific primers listed in Table S1. The vector constructs containing the native and mutated binding site were designated as wild-type (WT) and mutant (mut), respectively. Supplementary Table S2 provides information regarding the reference nucleotide positions within the 3’UTR, the restriction enzymes used, the position of the binding site, and the size of the reporter constructs.

### 2.4. Cell Line and Cell Culture

For our experiments, we employed the human embryonic kidney cell line HEK-293T, which was cultured in Dulbecco’s modified Eagle’s medium (Thermo Fisher Scientific, Waltham, MA, USA) supplemented with 10% fetal bovine serum (Biochrom, Cambridge, UK), 100 U/mL penicillin, and 100 U/mL streptomycin (Sigma, Burlington, MA, USA). The HEK-293T cells were maintained at 37 °C and 5% CO_2_ and subcultured after trypsinization with 1x Solution of 0.05% trypsin-EDTA (Thermo Fisher Scientific).

### 2.5. Automated Dual-Luciferase Reporter Assay

To enhance the reproducibility and replicability of results, we utilized the automatic liquid handling system epMotion 5075 (Eppendorf, Hamburg, Germany) for the Dual-Luciferase Reporter Assay in this study. Specifically, 3.6 × 10^4^ HEK-293T cells were seeded per well of the 96-well culture plates (Eppendorf) and were transfected 24 h later with 1 µL/well PolyFect transfection reagent (Qiagen, Hilden, Germany) containing 50 ng/well of each reporter vector and 200 ng/well of each expression vector. For each run, empty vector constructs (pMIR-empty and pSG5-empty) were included as a control, and all plasmid combinations were transfected in technical duplicates. After 48 h, cells were lysed and measured using the Dual-Luciferase Reporter Assay System manual with the GlowMax navigator microplate luminometer (Promega, Madison, WS, USA). We performed Luciferase assays of the WT 3’UTRs in four independent experiments and compared WT to mutated binding sites within the 3′UTR in three independent experiments. As a positive control, we re-evaluated the downregulation of four target genes (*PFKFB4, HMMR, UBQLN3,* and *ODF2*) previously studied by overexpressing microRNA-23a/b-3p using this automated technique, as shown in Figure S1.

### 2.6. RNAs Collection and Prepaartion

In this study, purified RNAs were obtained from a cohort of 82 men who were aged between 18 and 35 years old (with a mean age ± SD of 25.66 ± 4.21 years) [15]. This cohort included 41 subfertile men with oligoasthenozoospermia who sought infertility treatment at the IVF lab, as well as 41 age-matched normozoospermic control men with confirmed fertility. All participants underwent analysis of primary semen parameters according to the World Health Organization’s 2010 guidelines. It is worth noting that none of the individuals had abnormal cell morphology (<4%) or any known medical conditions that could cause infertility, such as Y chromosome microdeletions or chromosomal abnormalities. The detailed clinical characteristics of individuals are listed in Abu-Halima et al. [15].

### 2.7. cDNA Conversion, and qPCR 

Reverse transcription (RT) was conducted using 75 ng of total RNA in a 5 µL reaction volume, employing the Reverse Transcription Master Mix from Fluidigm Corporation, following the manufacturer’s instructions. Subsequently, 1.5 µL of cDNA was mixed with 1 µL of PreAmp Master Mix (Fluidigm Corporation, San Francisco, CA, USA) and 0.5 µL of pooled DELTAgene Assay Mix (500 nM each primer, Fluidigm Corporation) in a 5 µL reaction volume for pre-amplification. Exonuclease I treatment, as recommended by Fluidigm, was performed using a mixture of Exonuclease I (New England Biolabs, Ipswitch, MA, USA) and Exonuclease I Reaction Buffer added to the preamplification reaction. The preamplification process was then inactivated by incubation at 80 °C for 15 min. The samples were diluted with DNA suspension buffer and stored at −20 °C until RT-qPCR, following automation using the QIAcube™ Robotic Workstation (Qiagen).

For RT-qPCR of multiplex mRNA, the Biomark™ HD system from Fluidigm Corporation was employed. After preamplification, RT-qPCR was carried out using a 96.96 Dynamic Array™ IFC for Gene Expression (Fluidigm Corporation). The pre-amplified samples were mixed with SsoFast EvaGreen Supermix with low ROX (Bio-Rad Laboratories, Hercule, CA, USA) and DNA Binding Dye (Fluidigm), and the assay mix contained forward and reverse primers. The chip with the sample and assay mix was loaded into the Biomark™ HD system, and the specific thermal cycling protocol for mRNA analysis was performed. No-template controls (NTC) were included, and *GAPDH* was used for normalization. The data were analyzed using the Real-Time PCR Analysis Software (Fluidigm Corporation).

### 2.8. Statistical Analysis

GraphPad Prism 9 software from GraphPad Software in La Jolla, USA, and R Software from R Core Team in Vienna, Austria, was utilized for statistical analysis and visualization of the luciferase assay and RT-qPCR data. The ∆Ct values and fold change (FC) of each target gene were determined relative to *GAPDH*, which served as the endogenous control for RT-qPCR. The differences in ∆Ct values between oligoasthenozoospermic men and normozoospermic controls were assessed using Student’s unpaired two-tailed *t*-test. Spearman’s correlation was applied to evaluate the relationship between basic semen parameters and the expression levels of the target genes. The luciferase assay data were normalized to the empty expression vector (pSG5) and presented as mean ± standard error of the mean (SEM). *p*-values were calculated using Student’s unpaired two-tailed *t*-test, and for the wild-type experiments, Welch correction was applied. The levels of statistical significance were denoted as follows: * = 0.01 <  *p* ≤ 0.05; ** = 0.001 < *p* ≤ 0.01; *** = *p* ≤ 0.001.

## 3. Results

### 3.1. Target Gene Validation by Dual-Luciferase Reporter Assay

**Figure 1:**
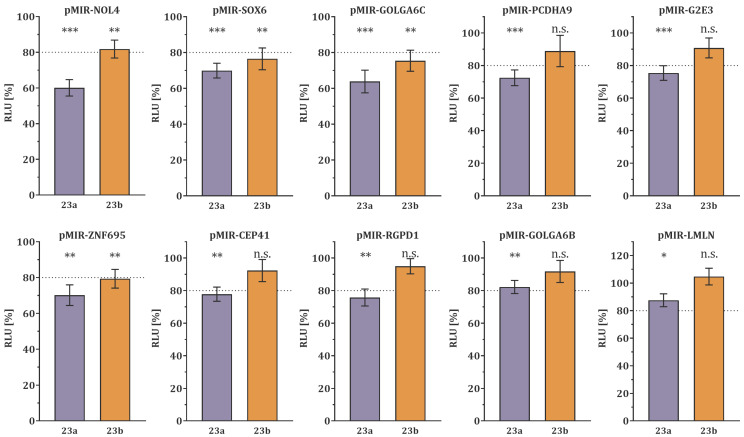
Dual-luciferase reporter gene assays were performed on the 3′ untranslated regions (UTRs) of ten genes, namely *NOL4, SOX6, GOLGA6C, PCDHA9, G2E3, ZNF695, CEP41, RGPD1, GOLGA6B,* and *LMLN.* HEK 293T cells were transfected with the reporter gene constructs (pMIR) and miRNA-expression plasmids (23a, pSG5-miR-23a-3p/ 23b, pSG5-miR-23b-3p) in the indicated combinations. The relative luciferase activity (RLU) was normalized to the empty control vectors (pMIR-empty and pSG5-empty). The results represent the mean of four independent experiments carried out in duplicates. The data are presented as mean ± SEM, and *p*-value was calculated using Welch *t*-test. A *p*-value of less than 0.05 was considered statistically significant (*** *p* < 0.001; ** *p* < 0.01; * *p* < 0.05, n.s. non-significant). Target genes with luciferase activity of less than 80% are considered high confidence target genes as indicated by the dotted line.

The dual-luciferase reporter assay confirmed the binding of microRNA-23a/b-3p within the 3′UTR of the 16 potential target genes. MicroRNA-23a-3p overexpression significantly decreased the luciferase activity of ten target genes (*NOL4*, *p* < 0.0001; *SOX6*, *p* = 0.0002; *GOLGA6C*, *p* = 0.0007; *PCDHA9*, *p* = 0.0007; *G2E3*, *p* = 0.0009; *ZNF695*, *p* = 0.0013; *CEP41*, *p* = 0.0014; *RGPD1*, *p* = 0.0023; *GOLGA6B*, *p* = 0.0031; *LMLN, p* = 0.0322), as depicted in Figure 1. Conversely, six target genes (*ZNF492, GNAT1, REEP1, CSNK1G1, FAM169A,* and, *TMEM215*, Figure S2) did not show a significant reduction in luciferase activity. Of the ten target genes with reduced luciferase activity, eight showed a high-confidence decrease of more than 20% (*NOL4*, 39.91%; *SOX6*, 30.11%; *GOLGA6C*, 36.19%; *PCDHA9*, 27.54%; *G2E3*, 24.60%; *ZNF695*, 29.83%; *CEP41*, 22.21%; *RGPD1*, 24.24%, Figure 1).

The overexpression of microRNA-23b-3p significantly decreased the luciferase activity of four target genes (*NOL4*, *p* = 0.0084; *SOX6*, *p* = 0.0062; *GOLGA6C*, *p* = 0.0041; *ZNF695*, *p* = 0.0056), as shown in Figure 1. Conversely, twelve target genes (*PCDHA9, G2E3, CEP41, RGPD1, GOLGA6B, LMLN, ZNF492, GNAT1, REEP1, CSNK1G1, FAM169A, TMEM215,* Figure 1, and Figure S1) did not show a significant reduction in luciferase activity. Three of the four target genes that had a decrease in luciferase activity by more than 20% were considered high-confidence target genes (*SOX6*, 23.54%; *GOLGA6C*, 24.61%; *ZNF695*, 20.65%).

### 3.2. Expression Level of MiR-23a/23b Target Genes in Spermatozoa by RT-qPCR

**Figure 2:**
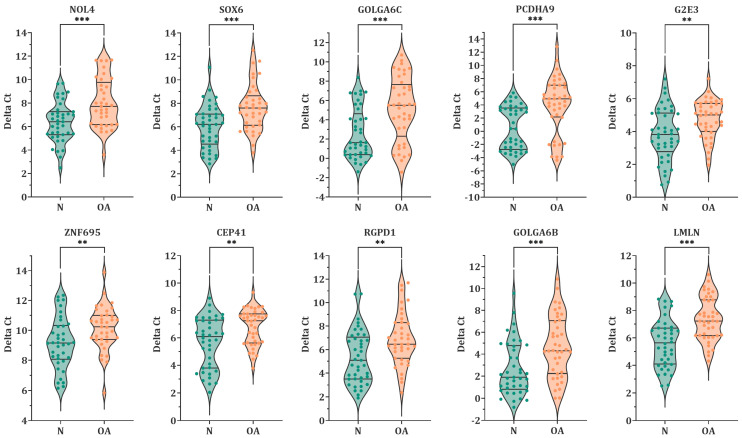
Violin plots display the expression levels of 10 chosen target genes in sperm samples from oligoasthenozoospermic men (n = 41) compared to normozoospermic controls (n = 41), as assessed by RT-qPCR. The data points are presented as ΔCt, and the plot lines depict the median and quartiles of ΔCt. The *p*-value was calculated using an unpaired two-tailed Student’s *t*-test, with a significance level set at *p* < 0.05 (*** *p* < 0.001; ** *p* < 0.01). N: normozoospermic controls, OA: oligoasthenozoospermic men.

To validate the effect of microRNA-23a-3p and microRNA-23b-3p on target genes, RT-qPCR analysis was performed on ten genes that showed a significant reduction in the luciferase activity. Results indicated that the expression level of all the ten target genes was significantly lower in oligoasthenozoospermic men compared to normozoospermic controls (Figure 2). Among these, four genes showed high statistical significance (*G2E3, p* = 0.0031, FC = 1.9; *ZNF695*, *p* = 0.0096, FC = 2.0; *CEP41*, *p* = 0.0023, FC = 2.2; *RGPD1*, *p* = 0.0031, FC = 2.9), whereas the remaining six genes were highly significant (*NOL4, p* = 0.0003, FC = 3.1; *SOX6, p* = 0.0003, FC = 3.2; *GOLGA6C, p* = 0.0002, FC = 5.9; *PCDHA9, p* = 0.0001, FC = 11; *GOLGA6B*, *p* = 0.0009, FC = 4.1; *LMLN*, *p* < 0.0001, FC = 3.4).

**Figure 3:**
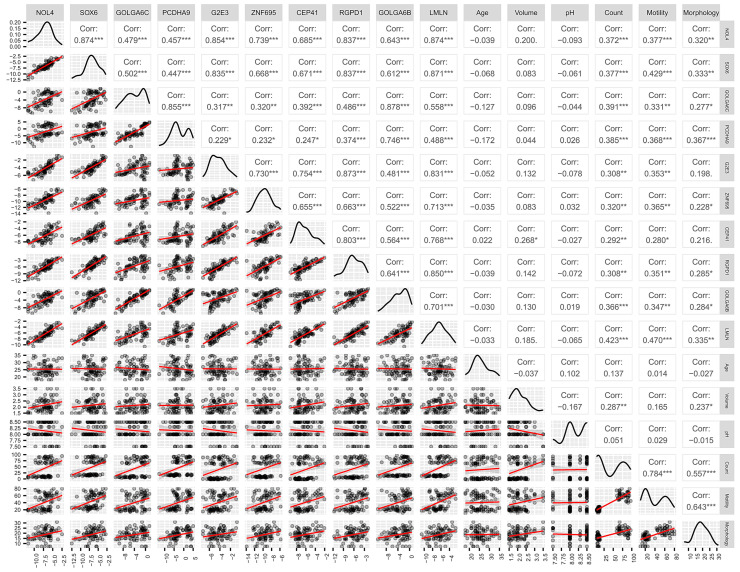
Correlation analysis of target gene abundance levels as determined by RT-qPCR (ΔCt) with age and basic semen parameters volume, pH, count, motility, and morphology. The lower and upper triangle show the scatter plot of the variables, the correlation coefficient and the significance. The diagonal shows the distribution of samples in density plots. Spearman correlation analysis and unpaired two-tailed *t*-test was performed. *p*-value < 0.05 was considered as statistically significant (*** *p* < 0.001; ** *p* < 0.01; * *p* < 0.05).

Figure 3 summarizes the correlation analysis between lower expression levels of the target genes and basic semen parameters. All ten target genes showed a weak to moderate positive correlation with sperm count and motility (*p*-value < 0.05), and eight genes (*NOL4, SOX6, GOLGA6C, PCDHA9, ZNF695, RGPD1, GOLGA6B,* and *LMLN*) showed a weak positive correlation with normal morphology of sperm. 

### 3.3. Validation of Binding-Site Specificity by Mutagenesis Dual-Luciferase Reporter Assay

**Figure 4:**
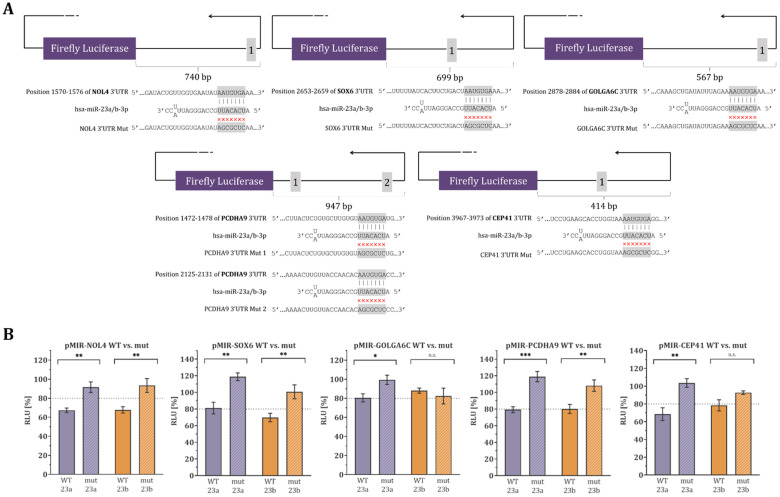
(**A**) Schematic diagram of the reporter vector constructs including microRNA-23a/b-3p binding sites. The localization of the predicted binding sites in the 3’UTRs of *NOL4, SOX6, GOLGA6C, PCDHA9,* and *CEP41,* the sequences of the microRNA-23a/b-3p binding sites, and the mutated binding sites (Mut) are shown [16]. (**B**) Mutagenesis dual-luciferase reporter gene assays of the 3′UTRs of *NOL4, SOX6, GOLGA6C, PCDHA9,* and *CEP41*. HEK 293T cells were transfected with the wild-type (WT) and mutated (mut) reporter gene constructs (pMIR) and the miRNA-expression plasmids (23a, pSG5-miR-23a-3p/23b, pSG5-miR-23b-3p) in the indicated combinations. The relative luciferase activity (RLU) was normalized to the empty control vectors (pMIR-empty and pSG5-empty). The results represent the mean of three independent experiments carried out in duplicates. Data are represented as mean ± SEM, and the *p*-value was calculated using an unpaired two-tailed Student’s *t*-test, and *p* < 0.05 was considered statistically significant (*** *p* < 0.001; ** *p* < 0.01; * *p* < 0.05; n.s. non-significant).

The mutagenesis dual-luciferase assay was used to determine the binding site specificity. The 3’UTR of eight target genes, which were high-confidence targets for at least one of the miRNAs (microRNA-23a-3p and microRNA-23b-3p) and contained binding sites complementary to the seed region of the miRNAs, were mutated. The wild-type and mutant constructs were then subjected to overexpression of microRNA-23a-3p or microRNA-23b-3p, and the relative luciferase activity was compared. For microRNA-23a-3p, the luciferase activity of the wild-type construct was significantly reduced compared to the mutant in five target genes (*NOL4, SOX6, GOLGA6C, PCDHA9,* and *CEP41*). Four of these target genes showed a reduction of more than 20% (*NOL4, SOX6, PCDHA9,* and *CEP41*), while GOLGA6C was borderline with an 18.95% reduction. The remaining three target genes (*G2E3, ZNF695,* and *RGPD1*) did not exhibit significant reductions in luciferase activity. Similarly, for microRNA-23b-3p, the luciferase activity of the wild-type construct was significantly reduced by more than 20% compared to the mutant in three target genes (*NOL4, SOX6*, and *PCDHA9*), and the wild-type construct of *PCDHA9* showed significant reduction only in this experiment. The remaining five target genes (*GOLGA6C, CEP41, G2E3, ZNF695,* and *RGPD1*) did not exhibit significant reductions in luciferase activity. Additionally, some of the target genes that were not significantly reduced in luciferase activity had mutants that showed reduced activity, indicating an off-target effect where the miRNA bound to a different binding site within the cloned fragment of the 3′UTR. Specifically, *G2E3* and *ZNF695* for microRNA-23a-3p, and *GOLGA6C, CEP41, G2E3, ZNF695,* and *RGPD1* for microRNA-23b-3p had reduced luciferase activity in their mutants. This suggests that the miRNAs may have additional targets beyond the identified ones. All these findings are summarized in Figure 4 and Figure S3.

## 4. Discussion

In this study, miRNA target genes were predicted using in silico analysis to identify 16 testis-specific potential target genes of microRNA-23a/b-3p, which we previously found to be more abundant in sperm from oligoasthenozoospermic men than normozoospermic controls [11,12]. These genes were cloned, and luciferase assays revealed that ten and four of them were target genes of microRNA-23a-3p and microRNA-23b-3p, respectively, with significantly reduced luciferase activity (Figure 1). To confirm the lower expression levels of these high-confidence target genes, RT-qPCR was conducted in sperm of oligoasthenozoospermic men compared to normozoospermic controls, and the results were positively correlated with the basic semen parameters (Figure 2 and Figure 3). To verify the binding-site specificity, we mutated the binding sites within the 3’UTR of these target genes and compared them to the wild type. Specifically, overexpression of microRNA-23a-3p and microRNA-23b-3p had an adverse effect on *NOL4, SOX6, GOLGA6C, PCDHA9*, and *CEP41*, as well as *NOL4, SOX6*, and *PCDHA9* in the mutant compared to the wild type (Figure 4). Using the single-cell type transcriptomic map from the Human Protein Atlas (proteinatlas.org), we found that *NOL4, SOX6, GOLGA6C, PCDHA9*, and *CEP41* were expressed in various stages of spermatogenesis, including spermatogonia, spermatocytes, early and/or late spermatids [17]. These genes are known to have testis-specific expression [17,18], and some of them have been implicated in physiological processes related to male reproduction. For example, SRY-Box Transcription Factor 6 (*SOX6*), which is a cancer-testis antigen with a high mobility group box, is directly associated with the sex-determining region Y (*SRY*) and is involved in various physiological and developmental processes [19,20,21,22]. *SOX6* has been identified as a co-factor of the testis-expressed centromere-associated protein (called Solt), which is essential for cell cycle progression and inactivation of cell division in mouse spermatogenesis [23,24,25]. Moreover, SOX6 is thought to play a role in sperm maturation and cycles during spermatogenesis in rodents [23,26]. However, the precise function of *SOX6* in human spermatogenesis remains unclear [23]. Golgin Subfamily A Member 6C (*GOLGA6C*) is predicted by the Gene Ontology consortium and the Human Protein Atlas to be involved in spermatogenesis, particularly in flagellum and Golgi organization, and was computationally identified as an interactor of *GSK3A*, which affects human sperm motility [17,27,28]. The Centrosomal Protein 41 kDa (CEP41) is localized on the primary cilia and plays a functional role in post-translational modifications, regulating the glutamylation of tubulins at the cilium [29]. Even if the relationship between *CEP41* ciliary function and sperm has not yet been explored, the Centrosomal Protein 192 (*CEP192*) is a well-known essential protein required for spermatogenesis. Recent evidence is emerging about “moonlighting” functions of cell division, i.e., meiosis players actively involving in spermatogenesis, which seems to be evolutionary conserved from *drosophila melanogaster* to human [30,31].

Regarding the mutant *G2E3, ZNF695*, and *RGPD1* 3’UTRs that demonstrated lower luciferase activities compared to the wild type (Figure S3), it is suggested that the miRNA may still activate the RNA interference (RNAi) mechanism. Previously, it was thought that only seed binding was essential, but now it is known that compensatory binding 3′ to the miRNA seed sequence also plays a role in miRNA binding [32,33]. Despite the mutated binding site being designed to prevent the binding of microRNA-23a/b-3p to the site, it is speculated that miRNA binding may have inadvertently shifted, causing single base pairing outside the seed and binding site. Other techniques including miR-CLIP can also be used to detect miRNA target interactions and these off-target effects [34]. 

It is noteworthy that even though microRNA-23a-3p and microRNA-23b-3p have identical seed sequences, there are differences in post-translational regulation strength, resulting in varying definitions of high-confidence target genes. MiRNAs with identical seed sequences typically belong to miRNA families, such as the miR-23 family [35,36]. While differences in target spectra and miRNA regulation strength of miRNA-family members using in vivo techniques are widely acknowledged, they are not well understood [37].

Our previous work indicated that there were differences in the targeting characteristics of microRNA-23a-3p and microRNA-23b-3p in sperm samples, as determined by RT-qPCR and luciferase assays. These findings are consistent with the results of the current study [11,12]. Previous research on other miRNA families has suggested a link between miRNA expression levels, MTI construction, and miRNA regulation strength and specificity [32,37]. It has been reported that supplementary base pairing outside the seed can influence the effectiveness of miRNA activity and the compensatory potential of imperfect base pairing within the seed [32,37]. We would like to emphasize that our study was not designed to identify differences in the functions of microRNA-23a-3p and microRNA-23b-3p. Further functional targeting experiments conducted in vivo will be necessary to fully comprehend the mechanisms of miRNA targeting.

We would like to acknowledge certain limitations of our study. An important aspect is the absence of fresh testicular biopsy samples and specific testicular cell lines for further analysis. Nonetheless, our findings revealed the impact of the selected microRNA-23a/b-3p on spermatogenesis and the testicular microenvironment. It is quite uncommon to obtain fresh testicular biopsies, particularly from fertile males. To some extent, we overcame this limitation by examining sperm samples, which represent the final product of spermatogenesis. Another limitation is that we did not validate the target gene protein levels in sperm samples obtained from subfertile men and healthy controls. We recently conducted a study of the proteomic landscape of human sperm obtained from oligoasthenozoospermic men, comparing these samples with samples from normozoospermic men using LC-MS/MS. Despite the detection of 4412 proteins and the analysis of 53 samples, we did not detect any of the miRNA-23a/b targets [38]. In the present study, we performed Western blot that also failed to detect the proteins that are encoded by the miRNA-23a/b target genes (Anti-NOL4, PA5-103986, Invitrogen and Anti-SOX6, ab64946, Abcam). The absence of miRNA-23a/b target proteins in our Western blot and mass spectrometry data might be attributed to the unique biology of sperm with its nearly silent transcription and translation processes [39]. It is widely recognized that certain mRNAs are remnants from earlier spermatogenesis events and that such miRNA can remain untranslated in sperm [39,40,41]. Other proteins that are transcribed during spermatogenesis can be removed as part of the removal of most cytoplasm during sperm development [42]. Additionally, proteins can be transported by extracellular vesicles that are frequently found in the seminal fluid [42]. These factors combined complicate the comparison of mRNAs and proteins in sperm. 

## 5. Conclusions

This study has affirmed that men with oligoasthenozoospermia exhibit reduced expression levels of specific genes such as *NOL4, SOX6, GOLGA6C, PCDHA9, G2E3, ZNF695, CEP41, RGPD1, GOLGA6B*, and *LMLN* when compared to men with normal sperm. Additionally, the study has identified particular interactions between microRNA-23a-3p and *NOL4, SOX6, GOLGA6C, PCDHA9*, and *CEP41*, as well as between microRNA-23b-3p and *NOL4, SOX6,* and *PCDHA9*. These findings suggest that the increased levels of microRNA-23a/b-3p and subsequent decrease in target gene expression are related to male subfertility, potentially by impacting key semen parameters. The study lays a foundation for future research focused on developing therapies for male infertility.

## Data Availability

The data presented in this study are available in the article and Supplementary Materials.

## Supplementary Materials

The following supporting information can be downloaded at: https://www.mdpi.com/article/10.3390/biology12060800/s1, Figure S1: Dual-luciferase reporter gene assays of previously tested genes *PFKFB4, HMMR, UBQLN3*, and *ODF2*. HEK 293T cells were transfected with the reporter gene constructs (pMIR), and the miRNA-expression plasmids (23a, pSG5-miR-23a-3p/23b, pSG5-miR-23b-3p) in the indicated combinations. The relative luciferase activity (RLU) was normalized to the empty control vectors (pMIR-empty and pSG5-empty). The results represent the mean of four independent experiments carried out in duplicates. Data are represented as mean ± SEM, and *p*-values were calculated using Welch *t*-test, and *p* < 0.05 was considered statistically significant (*** *p* < 0.001; ** *p* < 0.01; * *p* < 0.05; n.s. non-significant). Figure S2: Dual-luciferase reporter gene assays of the 3′UTRs of *ZNF492, GNAT1, REEP1, CSNK1G1, FAM169A,* and *TMEM215* that were not significantly reduced in luciferase activity. HEK 293T cells were transfected with the reporter gene constructs (pMIR), and the miRNA-expression plasmids (23a, pSG5-miR-23a-3p/ 23b, pSG5-miR-23b-3p) in the indicated combinations. The relative luciferase activity (RLU) was normalized to the empty control vectors (pMIR-empty and pSG5-empty). The results represent the mean of four independent experiments carried out in duplicates. Data are represented as mean ± SEM, and the *p*-value was calculated using Welch *t*-test. *p* < 0.05 was considered statistically significant (*** *p* < 0.001; ** *p* < 0.01; * *p* < 0.05; n.s. non-significant). Figure S3: Mutagenesis dual-luciferase reporter gene assays of the 3′UTRs of *G2E3, ZNF695,* and *RGPD1* that were not significantly reduced in luciferase activity comparing wild-type (WT) and mutated (mut) 3’UTR. HEK 293T cells were transfected with the WT and mut reporter gene constructs (pMIR), and the miRNA-expression plasmids (23a, pSG5-miR-23a-3p/ 23b, pSG5-miR-23b-3p) in the indicated combinations. The relative luciferase activity (RLU) was normalized to the empty control vectors (pMIR-empty and pSG5-empty). The results represent the mean of three independent experiments carried out in duplicates. Data are represented as mean ± SEM, and the *p*-value was calculated using unpaired two-tailed Student’s *t*-test. *p* < 0.05 was considered statistically significant (*** *p* < 0.001; ** *p* < 0.01; * *p* < 0.05; n.s. non-significant). Table S1: Sequences of cloning and mutagenesis primers. Table S2: Constructs with NCBI Reference Sequence, restriction enzymes for cloning, nucleotide position in 3′UTR, binding site position, and size of the amplified fragment. Table S3: Sequences of RT-qPCR primers and their respective design reference sequence.
