# Supplementary material for: Towards a More Comprehensive Picture of the MicroRNA-23a/b-3p Impact on Impaired Male Fertility
Source: Biology (Basel). 2023 May 31;12(6):800. doi: 10.3390/biology12060800 (PMC10294816; doi:10.3390/biology12060800)
Supplement: Supplementary file 1 [file biology-12-00800-s001.zip › biology-2378650-supplementary/Supplementary Tables.pdf]

Supplementary Tables

Supplementary Table S1: Sequences of cloning and mutagenesis primers.

Target Gene	Sequence<br>(restriction sites SpeI and SacI are in underline bold and mutated sites are shown in bold and italic)
CEP41_for	GG**ACTAGT**GGGTAGTAATACTTTCCTAG
CEP41_rev	C**GAGCTC**GGTGTGTGACCTTTGCGTGC
CEP41_for_mut	CCTGAAGCACCTGGTAA***AGCGCTC***GGCAAAGAGAGGCC
CEP41_rev_mut	GGCCTCTCTTTGCC***GAGCGCT***TTACCAGGTGCTTCAGG
GOLGA6C_for	GG**ACTAGT**GGAATGATCTTCAGCTAGCC
GOLGA6C_rev	C**GAGCTC**CTATATTATGCATAGGGCTG
GOLGA6C_rev_mut	C**GAGCTC**CTATATTATGCATAGGGCTGATTTATATTT***GAGCGCT***TCTAAATATCAGC
GOLGA6B_for	GG**ACTAGT**CGGACAATAATGTGTTCATC
GOLGA6B_rev	C**GAGCTC**GCAGTTACACTTATGAATAG
GOLGA6B_for_mut	CCAAAGCTGATATTTAGAA***AGCGCTC***AAATGTAAATCAGCCC
GOLGA6B_rev_mut	GGGCTGATTTACATTT***GAGCGCT***TTCTAAATATCAGCTTTGG
G2E3_for	GG**ACTAGT**GATATTTGAATGATCTCTGTGG
G2E3_rev	C**GAGCTC**GGTATGTTAGTGTACAGATTG
G2E3_for_mut	CAAAGTGCTAAC***AGCGCTC***AAATATATAGTCCC
G2E3_rev_mut	GGGACTATATATTT***GAGCGCT***GTTAGCACTTTG
NOL4_for	GG**ACTAGT**GCACTCTTGTGTGTAG
NOL4_rev	C**GAGCTC**CAGAGAAAATTATACCAGCCC
NOL4_for_mut	GTGATACTGTTGGTGAATAT***AGCGCTC***AAAATCTCATTGAAATATGAG
NOL4_rev_mut	CTCATATTTCAATGAGATTTT***GAGCGCT***ATATTCACCAACAGTATCAC
PAPOLB_for	GG**ACTAGT**GCTATCAAAGCTCCATATTC
PAPOLB_rev	C**GAGCTC**GCAAGGCTAATTGGAAAGCC

**PAPOLB_rev_mut**	C**GAGCTC**GCAAGGCTAATTGGAAAGCCTGTTAAGTTTGCTGTCTAGATTAAGAAAGACTCAACTTTCATTCATTCACATCTGTTGCCCC***GAG<br>CGCT***TCC
**PCDHA9_for**<br>**PCDHA9_rev**<br>**PCDHA9_for_mut**<br>**PCDHA9_rev_mut_1**<br>**PCDHA9_rev_mut_2**	GG**ACTAGT**GAGCAGATTTTTCAATCTAC<br>C**GAGCTC**GATTCTCCTAGTTCAAAAGGC<br>CTCTGTGCTTGTGT***AGCGCTC***TGTTAATGCAACTATTACC<br>GGTAATAGTTGCATTAACA***GAGCGCT***ACACAAGCACAGAG<br>C**GAGCTC**GATTCTCCTAGTTCAAAAGGCACGTAAGGTCAGATACATGGGCTTCAGTGGGGCAAAATGATCACAACCTACAATGTAGCAATG<br>G***GAGCGCT***GTG
**RGPD1_for**<br>**RGPD1_rev**<br>**RGPD1_rev_mut**	GG**ACTAGT**GAGATCACTTGATGATAC<br>C**GAGCTC**CCAAACAAAAAAGAGCTTTG<br>C**GAGCTC**CCAAACAAAAAAGAGCTTTGTTTCTTT***GAGCGCT***CATTTCTC
**SOX6_for**<br>**SOX6_rev**<br>**SOX6_for_mut**<br>**SOX6_rev_mut**	GG**ACTAGT**CCTCTCCTCCTCAGTTCACTC<br>C**GAGCTC**GGATATTTAGAGCCAAACTTGGC<br>GGTATTTTTTATCACTTCTGACT***AGCGCTC***AACTGTTGTACG<br>CGTACAACAGTT***GAGCGCT***AGTCAGAAGTGATAAAAAATACC
**ZNF695_for**<br>**ZNF695_rev**<br>**ZNF695_for_mut**<br>**ZNF695_rev_mut**	GG**ACTAGT**CCTATTCACAACTTCACAGC<br>C**GAGCTC**AGAGATGAGGTTTTGCCATG<br>CTGTTTTACAGATGCAGTA***AGCGCTC***AAAAATGTCTAATC<br>GATTAGACATTTTT***GAGCGCT***TACTGCATCTGTAAAACAG
**CSNK1G1_for**<br>**CSNK1G1_rev**	GG**ACTAGT**CCTCCAGGAGAGATATTTGTGC<br>C**GAGCTC**GAAGTGACTCACAGGAGAAG
**FAM169A_for**<br>**FAM169A_rev**	GG**ACTAGT**GACCATTGGGGGCTAACAG<br>C**GAGCTC**GACTAGATGTTTTCCTTACC
**GNAT1_for**	GG**ACTAGT**GGCACACTCACCTTGGGTGC

GNAT1_rev	C**GAGCTC**GAGATTTGTACACCCATGTTC
LMLN_for<br>LMLN_rev	C**GAGCTC**CAAGAAAGTCTCACTCTGTCA<br>G**GACTAG**TGCTCAACCTGCGTAATGGTC
REEP1_for<br>REEP1_rev	GG**ACTAGT**GCTTACACAGCTTCTTAGC<br>C**GAGCTC**GCCTGTGTCTGTAAGTCAGC
TMEM215_for<br>TMEM215_rev	GG**ACTAGT**CTTCTTACACCTGAGTTTCC<br>C**GAGCTC**GAGACAGAATGATATAAAGG
ZNF492_for<br>ZNF492_rev	GG**ACTAGT**GGTGAGAAATAATAGAAATATG<br>C**GAGCTC**CATTTACAGGGTTTTTCTTCG

Supplementary Table S2: Constructs with NCBI Reference Sequence, restriction enzymes for cloning, nucleotide position in 3'UTR, binding site position, and size of amplified fragment

Construct	NCBI Reference Sequence	Restriction enzymes for cloning	Nucleotide position in 3'UTR	Binding site position in 3'UTR	Size of amplified fragment
pMIR-CEP41	NM_001257158.2	SpeI - SacI	3688 - 4401	3967 - 3973	714 bp
pMIR-GOLGA6C	NM_001164404.2	SpeI - SacI	2348 - 2914	2878 - 2884	567 bp
pMIR-GOLGA6B	NM_018652.5	SpeI - SacI	2391 - 2984	2883 - 2889	593 bp
pMIR-G2E3	NM_001308097.2	SpeI - SacI	934 - 1637	1262 - 1268	704 bp
pMIR-NOL4	NM_001198546.1	SpeI - SacI	963 - 1702	1570 - 1576	750 bp
pMIR-PAPOLB	NM_020144.5	SpeI - SacI	937 - 1604	1516 - 1522	668 bp
pMIR-PCDHA9	NM_031857.2	SpeI - SacI	1270 - 2216	1472 - 1478, 2125 - 2131	947 bp
pMIR-RGPD1	NM_001024457.4	SpeI - SacI	920 - 1398	1365 - 1371	497 bp
pMIR-SOX6	NM_001145811.2	SpeI - SacI	2293 - 2991	2653 - 2659	699 bp
pMIR-ZNF695	NM_020394.5	SpeI - SacI	44 - 716	400 - 406	673 bp
pMIR-CSNK1G1	NM_001329605.2	SpeI - SacI	8 - 418	145 - 151	418 bp
pMIR-FAM169A	NM_015566.3	SpeI - SacI	2262 - 2965	2593 - 2599	704 bp

pMIR-GNAT1	NM_144499.3	SpeI - SacI	1715 - 2428	2167 - 2173	714 bp
pMIR-LMLN	NM_001136049.3	SpeI - SacI	2935 - 3720	3379 - 3385	786 bp
pMIR-REEP1	NM_001164730.2	SpeI - SacI	2077 - 2576	2482 - 2488	500 bp
pMIR-TMEM215	NM_212558.3	SpeI - SacI	1616 - 2323	1975 - 1981	708 bp
pMIR-ZNF492	NM_020855.3	SpeI - SacI	-12 - 468	11 - 17	480 bp

Supplementary Table S3: Sequences of RT-qPCR primers and their respective Design Reference Sequence.

Target gene	Forward Primer	Reverse Primer	Design RefSeq
CEP41	AACCCGTGAGGCTAGAACC	TGGGTTCTGTGGTATCCTTTTCA	NM_018718.N
G2E3	TTGCAAAGAAGGGCGAGACTA	TGCCACTGGAACCACAACA	NM_017769.N
GOLGA6B	CTCCACCGGCGGTTACA	TCGCTCTAGCTGGACTTGTTTTA	NM_018652.N
GOLGA6C	GCTGAACGCACACGTGAC	TTTAGCATATTCGTCCCGCTCTA	NM_001164404.N
LMLN	AGCTCTGTCCTCCAGAAACA	GCTCGAGGAACAGGAACAAA	NM_001136049.N
NOL4	GGAACGAGAGGCGAGAGAA	TCCTCAGCTCCTCGGTCTA	NM_001353233.N
PAPOLB	AGCCTGCCATTTCTCCATCA	AGGTCTGGATGGCTTATGAGAC	NM_020144.N
PCDHA9	ACGCTCTCGGTTCCTGTAAA	TCTGCGTCTAGGTCGATCAC	NM_014005.N
RGPD1	AGACCGCTTAGCATGGACTA	TTCTTTAGCTTCATAATACAGCTTTGC	NM_001024457.N
SOX6	CAGCAGCAACTTCTGCAACA	TGAGCGGAGGCATGTGAC	NM_001367873.N
ZNF492	ACCTGTCTGGAGCAAGGAAA	GGTCTCGGGCAAAATAAGAACA	NM_020855.N
ZNF695	CCCTTGGTGAGGATAGCTTCAA	TCTTTCCTTGCCTCCAGACA	NM_020394.N
ACTB	CCAACCGCGAGAAGATGAC	TAGCACAGCCTGGATAGCAA	NM_001101.N
GAPDH	GAGAACGGGAAGCTTGTCATCA	TGGACTCCACGACGTACTCA	NM_002046.
