# Supplementary figures and images for: Towards a More Comprehensive Picture of the MicroRNA-23a/b-3p Impact on Impaired Male Fertility
Source: Biology (Basel). 2023 May 31;12(6):800. doi: 10.3390/biology12060800 (PMC10294816; doi:10.3390/biology12060800)

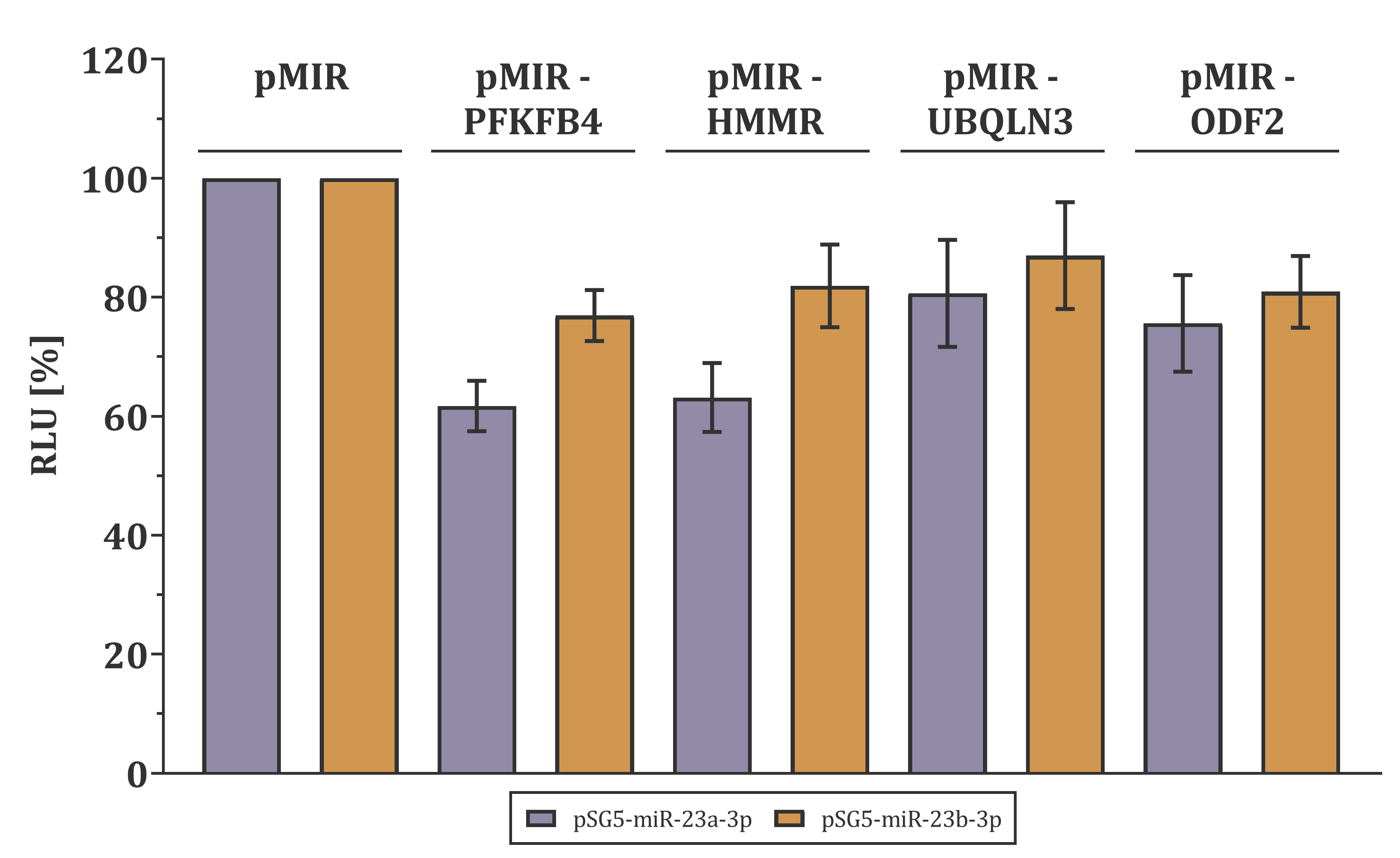

Supplement: Supplementary file 1 [file biology-12-00800-s001.zip › biology-2378650-supplementary/Supplementary Figure 1.tif]

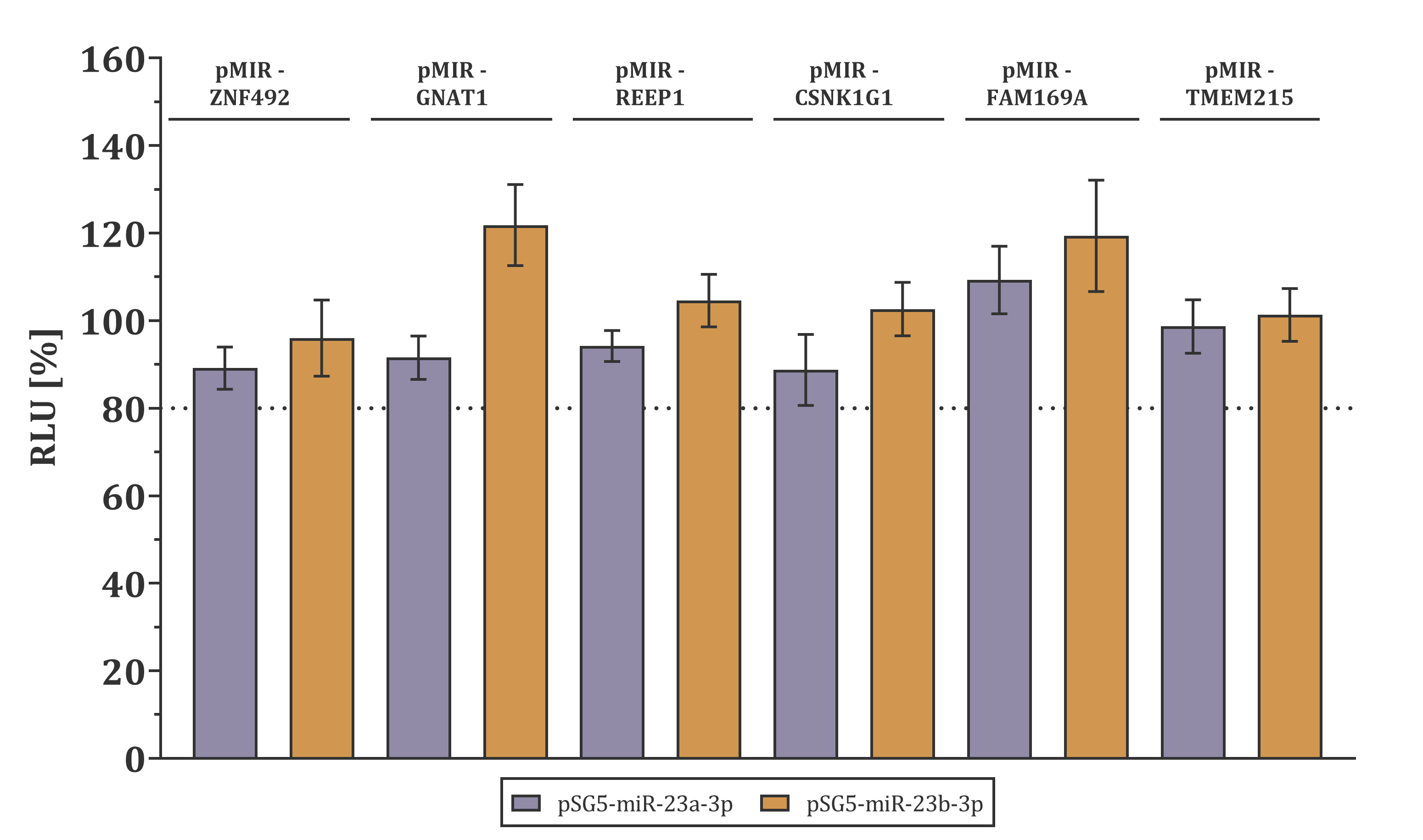

Supplement: Supplementary file 1 [file biology-12-00800-s001.zip › biology-2378650-supplementary/Supplementary Figure 2.tif]

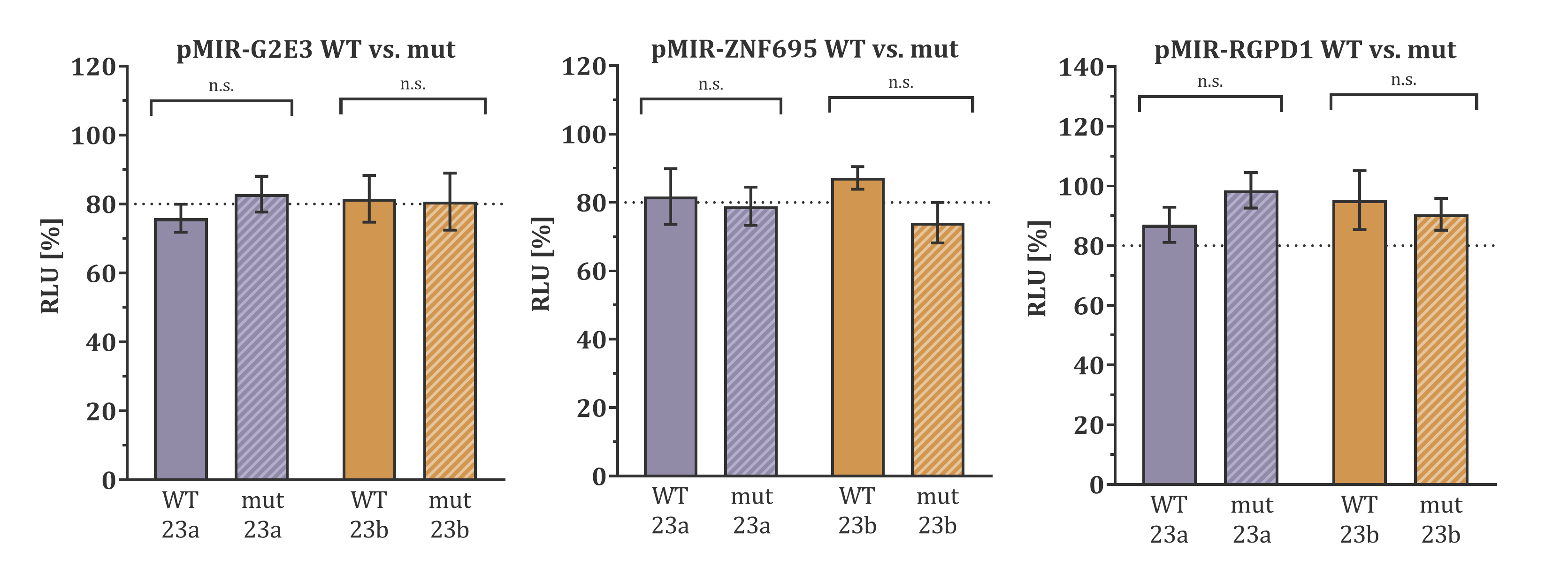

Supplement: Supplementary file 1 [file biology-12-00800-s001.zip › biology-2378650-supplementary/Supplementary Figure 3.tif]
